# Strategic insights from a Delphi study: enhancing employment for multiply marginalized people with disabilities

**DOI:** 10.3389/fresc.2024.1443302

**Published:** 2024-09-04

**Authors:** Kelsey S. Goddard, Noelle K. Kurth, Jean P. Hall, Lyndsie M. Koon, Corey L. Moore, Kacie R. Dentleegrand

**Affiliations:** ^1^ Institute for Health and Disability Policy Studies, University of Kansas, Lawrence, KS, United States; ^2^ Department of Rehabilitation and Disability Studies, Langston University, Langston, OK, United States

**Keywords:** multiply marginalized populations, disability, employment, Delphi study, DEI (or diversity equity and inclusion)

## Abstract

**Introduction:**

The employment landscape for multiply marginalized people with disabilities presents significant challenges, exacerbated by intersecting identities such as race/ethnicity, sexual orientation, gender identity, poverty, and geography. Recent studies highlight the compounded employment disparities faced by this group, including discriminatory hiring practices, inadequate accommodations, and uneven gains in employment during the COVID-19 public health emergency.

**Methods:**

Our study employed a three-round Delphi process with 20 diverse experts across 14 states across the United States (U.S.) to formulate recommendations for improving employment experiences for multiply marginalized people with disabilities. The panel's insights were gathered through surveys administered online, with each round designed to refine the collective recommendations. This iterative process aimed to build a consensus on the most effective policy and practice recommendations for improving employment outcomes within this population.

**Results:**

The Delphi study identified key areas for strategic focus, including emergency preparedness, education and training, transportation, assistive technology, workplace accommodations, and combating discrimination and stigma. Notable recommendations included improving emergency preparedness training, enhancing employment education, increasing funding for accessible transportation and assistive technology, and promoting inclusive hiring practices. The study also emphasized the need for policies supporting telework and simplifying disability-related benefits.

**Discussion:**

The findings highlight the critical role of tailored strategies to address employment challenges faced by people with disabilities from marginalized communities. Meaningfully and fully implementing these recommendations would create a more inclusive environment that improves employment outcomes for multiply marginalized people with disabilities.

## Introduction

Despite many years of work and dedicated funding, the employment landscape for multiply marginalized people with disabilities remains fraught with significant challenges that hinder participation in the workforce (1–3). These individuals experience a unique set of barriers, as their employment prospects are not only influenced by their disability but also by intersecting factors such as race/ethnicity, sexual orientation, gender identity, poverty status, and geographic location. Recent studies, such as those by Moore et al. (4), Manyibe et al. (5), and Webb et al. (6), emphasize that such intersecting identities exacerbate employment disparities, with multiply marginalized people with disabilities facing higher rates of unemployment and underemployment compared to their non-disabled counterparts or those with a single axis of marginalization. Specific challenges for multiply marginalized people with disabilities include disproportional experiences of workplace harassment, discriminatory hiring practices, and inadequate workplace accommodations (7). Furthermore, a literature review by Shaw et al. (8) underlines the ongoing issues with workplace accessibility and job adaptability, while Burke et al. (9) point to societal misconceptions about the capabilities of people with disabilities, impacting their employment opportunities and career progression. Other research highlights the lack of disability knowledge among employment services staff and employers as a major barrier to employment of people with disabilities (10, 11).

These challenges culminate in the underemployment of individuals with disabilities, as demonstrated by Brooks (12), who analyzed employment rates among U.S.-born adults aged 21–65, considering both disability and racial or ethnic background. Brooks's findings reveal a clear employment hierarchy: 79% of white non-disabled individuals are employed, the highest rate among the groups studied, followed by 76% of non-disabled Hispanics/Latines, and 69% of non-disabled African Americans. When disability is factored in, the employment rates drop significantly, illustrating a stark disadvantage for disabled individuals across all racial and ethnic groups. Only 36% of disabled white individuals and 34% of disabled Hispanics/Latines are employed, while just 23% of disabled African Americans have jobs, marking them as the most disadvantaged group in terms of employment.

The early stages of the COVID-19 pandemic further amplified these challenges, introducing unprecedented disruptions in the job market, which disproportionately affected vulnerable populations, including multiply marginalized people with disabilities. Research conducted during the pandemic illustrated how the shift to remote work, while beneficial for some, deepened the digital divide and accessibility issues, posing additional hurdles for this group (13). Furthermore, the economic downturn triggered by the pandemic significantly affected the employment of multiply marginalized people with disabilities, with a substantial portion experiencing job losses, layoffs, and reduced employment opportunities (14). While people with disabilities as a group have more recently experienced record high employment rates, those from racial and ethnic minorities still experience greater rates of unemployment (15, 16).

In this context, there is a critical need for a nuanced understanding of the evolving employment challenges faced by individuals with disabilities from other marginalized communities and for developing strategies that are responsive to their specific needs. Addressing these challenges requires a concerted effort from policymakers, job center staff, vocational rehabilitation and education professionals, industry leaders, and community advocates to foster an inclusive employment environment that is adaptable to the changing economic landscape. To this end, the current study utilized a three-round Delphi process to engage a panel of experts and vested parties to forecast strategies for improving the future of employment statuses among multiply marginalized people with disabilities. The Delphi method (17), known for its systematic consensus-building approach, is particularly appropriate for exploring complex and multifaceted issues such as employment challenges in the post-public health emergency era. By leveraging the collective expertise of the panel, this study aimed to generate actionable insights that will inform policy and practice, ultimately contributing to the creation of a more inclusive and equitable employment environment for this underserved and multiply marginalized population.

## Methods

### Study design & sample

This study utilized a three-round Delphi process, tailored to engage a panel of experts in a structured series of surveys to forecast and strategize on the employment challenges and opportunities for multiply marginalized people with disabilities. The Delphi process was conducted in from August to December 2023, with each round designed to build upon the insights and feedback from the previous one, thereby refining and honing the panel's collective recommendations.

The panel consisted of 20 key participants from 14 U.S. states, representing both rural and urban geographical areas. Recruitment was designed to include multiply marginalized people with disabilities, employers, disability advocates, and public employment service (PES) providers. Panelists were nominated by members of a larger research center team. A team of three researchers then reviewed a list of 61 referrals and independently highlighted individuals based on diverse demographic characteristics. The researchers selected panelists for whom there was the most consensus among their independent highlighted lists. These participants were chosen based on their direct experience, potential ability to inform policy and practice recommendations, and diverse perspectives on the employment of multiply marginalized individuals with disabilities. This selection process helped to support the inclusion of valuable insights from different sectors.

Participants ultimately included professionals in higher education; at centers for independent living (CILs); Vocational Rehabilitation (VR), including tribal VR and VR services for the blind; mental health and substance abuse; occupational therapy (OT); and career and technical training. Their inclusion ensured a wide-ranging understanding of the employment barriers faced across various geographic and community contexts.

With an average of nearly 20 years of experience in working with people with disabilities, the panelists’ backgrounds were notably diverse, spanning different genders—including female, male, non-binary and transgender—and ages ranging from the mid-30s to early 60s. Forty percent of participant experts identified as having a disability themselves and, in two cases, utilized disability employment services in their own efforts to find work. The inclusion of these personal perspectives added value and validity to the findings. This rich mix of professional expertise and personal experiences was deliberate to facilitate diverse and comprehensive recommendations. Detailed demographics of the panelists are presented in Table 1.

**Table 1:** Delphi study participant demographics, *n* = 20.

	%	*n*
Age, range = 36–61; Mean = 51.1		
Yrs. of experience, range = 2–35; Mean = 19.9 yrs.		
Has a disability	40.0	8
Disability type, self-categorization		
Mental illness/psychiatric	10.0	2
Physical/mobility	15.0	3
Chronic illness/disease	5.0	1
Developmental	5.0	1
Sensory	5.0	1
Race/ethnicity		
American Indian/Alaskan Native	10.0	2
Black/African American	20.0	4
Hispanic/Latine	10.0	2
White	50.0	10
Multi-Racial	5.0	1
Prefer not to answer	5.0	1
Gender identity		
Female	65.0	13
Male	25.0	5
Non-binary, transgender	10.0	2
Sexual identity		
Straight/Heterosexual	70.0	14
Gay/Lesbian/Homosexual/Bisexual	10.0	2
Prefer not to answer	20.0	4
Geographic area currently serving		
Urban only	25.0	5
Both urban and rural	75.0	15

To minimize attrition, we maintained consistent communication with participants throughout the study, offering reminders and support to encourage completion. Despite these efforts, some attrition did occur over the course of the three rounds. Starting with 20 participants in Round 1, there were two individuals who were unable to complete Rounds 2 and 3 and an additional participant who did not complete Round 3 leaving a final sample size of 17. This Attrition occurred due to non-response from these participants in Rounds 2 and 3 of the study.

### Data collection and measurement techniques

The Delphi study was executed in a sequential, three-round approach to gain expert insights on the employment challenges and strategies for multiply marginalized people with disabilities.

In Round 1, experts engaged in a qualitative exploration, articulating their thoughts on pertinent policy and practice recommendations. During this round, Delphi participants were asked to recommend policy and practice solutions across eight factors: (1) Education and Training, (2) Transportation, (3) Assistive Technology (AT), (4) Accommodations, (5) Discrimination and Stigma, (6) Telework, (7) Changes in the Employment Landscape after COVID-19, and (8) Employer Incentives to Hire People with Disabilities. These initial eight factors were informed by a team of researchers with extensive experience in employment for multiply marginalized people with disabilities, serving as a starting point for discussion. Participants were also given the opportunity to suggest their own categories. As a result, four new panel-generated factors were included: Complex Rules Surrounding Disability-Related Benefits; Emergency Preparedness Training; Poverty, Scarcity, and Financial Empowerment; and Vocational Rehabilitation (VR) Eligibility Requirements. Participants were provided with additional details and probing questions via open-ended items across the eight factors and any added by the individual participant. For each factor, participants were asked to think of (a) reasons why people with disabilities from marginalized groups do not have equitable access and (b) what changes to both policy and practice they would suggest to address the inequity. This initial round captured a diverse range of perspectives, setting a solid foundation for the subsequent two rounds.

In Round 2, the qualitative data gathered from the open-ended items in Round 1 were distilled into statements. Delphi participants then evaluated these items, rating them on a 1 to 5 scale based on their overall perceived importance in terms of the work needed to improve employment outcomes for multiply marginalized people with disabilities over the next 5 years. Participant ratings of each recommendation within all factors, aided in the prioritization of the issues and recommendations identified previously.

Round 3, the final round of the Delphi, served to achieve consensus among the experts. In this round, the previously rated recommendations were provided to the panelists along with their average ratings across all participants. Panelists could revise their ratings based on the group scores or choose to leave them the same. Final ratings were then determined for each recommendation and overall factor.

This three-round Delphi process sought to provide insightful and actionable recommendations to inform and guide future employment policies and practices for multiply marginalized people with disabilities.

To mitigate the potential for consensus bias, where participants may gravitate towards prevailing opinions, several measures were implemented throughout the Delphi process. Firstly, all rounds were conducted anonymously from other panelists, and open-ended questions were included to capture a wide range of insights. The iterative nature of the Delphi process, where participants reviewed and refined their feedback across multiple rounds, further allowed for the integration of diverse perspectives. Panelists were encouraged to provide detailed explanations for their ratings and suggestions, ensuring that all perspectives were documented. The diverse composition of the expert panel, encompassing various cultural, socioeconomic, and geographical backgrounds, naturally counteracted consensus bias. Facilitators actively monitored feedback to ensure all perspectives were included, integrating opinions from all panelists into the final consensus.

All feedback was collected using the Qualtrics survey platform, which is fully accessible, and was chosen for its compatibility with screen readers, adjustable text sizes, and alternative text for images. Additionally, participants had the option to request the survey in alternative formats, such as paper copies or via telephone interviews, to ensure accessibility for all participants.

All study procedures and protocols were approved by the University of Kansas Institutional Review Board (IRB, Study #00150506). Participants provided informed consent before engaging in the study, ensuring they were aware of the study's purpose, procedures, and their right to withdraw at any time.

### Data analysis

The analysis of the Delphi study data employed a blend of qualitative and quantitative methods to extract meaningful insights. In the first round, qualitative data were subjected to thematic analysis, a process which involves systematically identifying, analyzing, and reporting patterns (themes) within the data. This analysis was conducted by one researcher who read through the responses multiple times to become familiar with the content, then coded the data to identify key themes and insights. These themes provided a rich understanding of the perspectives shared by the experts regarding the employment of multiply marginalized persons with disabilities.

Following the initial thematic analysis, two additional researchers engaged in a rigorous review of the themes that emerged from each of the 12 factors. To ensure the accuracy and reliability of the findings, these researchers independently examined the themes and associated recommendations, validating or challenging the initial researcher's synthesis. This step included reviewing the coding process, comparing interpretations, and discussing any discrepancies. This collaborative review process was iterative, involving multiple rounds of discussion and refinement until a consensus was reached on the key themes and insights.

For quantitative analyses in the subsequent rounds, statistical methods were applied to determine mean and median scores and observe changes in rankings. This approach was instrumental in pinpointing areas of consensus and highlighting the recommendations deemed most important by the panelists. SPSS version 28 was used for all quantitative analyses.

## Results

The Delphi study's iterative process facilitated a broad exploration of strategies to enhance employment opportunities for people with disabilities, focusing on identifying and prioritizing strategies to address the employment challenges faced by multiply marginalized persons with disabilities. Table 2 provides the relative rating of each factor's importance, and Table 3 provides the top 3–5 ranking recommendations within each of those factors.

**Table 2:** Relative importance of factors related to employment for people with disabilities from multiply marginalized groups, greatest to least important^a^.

Factor	Median
1. Emergency preparedness training^b^	4.118
2. Education and training	4.029
3. Assistive Technology (AT)	4.000
3. Accommodations	4.000
3. Complex rules surrounding disability-related benefits^b^	4.000
3. Poverty, scarcity and financial employment^b^	4.000
4. Discrimination and stigma	3.941
4. Telework	3.941
4. VR eligibility requirements^b^	3.941
5. Transportation	3.906
6. Employer incentives to hire people with disabilities	3.824
7. Changes in the employment landscape due to COVID-19	3.558

^a^ 1–5 scale with 5 = greatest importance and 1 = least importance.

^b^ Factor added by Delphi participants, others provided by researchers.

**Table 3:** Top 3–5 recommendations by factor.

Emergency Preparedness Training^a^
• Provide regular training for employees, including emergency response teams, on assisting people with disabilities during evacuations.
• Focus on the need for proper emergency evacuation plans and protocols that are safe and consider the varied needs of people with disabilities, including those from marginalized communities.
• Establish committees comprised of diverse representatives, including people with disabilities, to oversee and implement inclusive emergency plans.
Education and Training
• Encourage the hiring of Human Resources (HR) professionals who are knowledgeable about disability issues and open to alternative qualification assessment methods, such as portfolios.
• Provide more skills-orientated employment education in schools, recognizing that not all students need a college education to succeed and that exposure to alternative employment opportunities.
• Provide incentives for transportation to education and training opportunities, especially in rural communities (e.g., Free Fare programs).
Assistive Technology (AT)
• Allocate more funding to AT Programs to provide information to employers and employees regarding current AT that is available and the importance of providing access to assistive technology for people with disabilities in the workplace.
• Promote the integration of AT in school and job training programs to increase exposure and success in post-secondary education and employment.
• Ensure that information about reasonable accommodations and assistive technology is readily accessible to all employees as standard practice.
• Mandate coordination between state AT Programs and state VR programs, requiring state AT programs to be a partner in Workforce Innovation and Opportunity Act (WIOA) state plans.
Accommodations
• Ensure VR counselors discuss disability accommodations with people from marginalized groups during the VR process, enabling informed choices and self-advocacy.
• Offer incentives or grants to offset the costs of accommodations for employers.
• Provide funds to support teaching people with disabilities to advocate for themselves and for reasonable accommodations while looking for a job and once in a job.
Complex Rules Surrounding Disability-Related Benefits^a^
• Raise income and asset caps for SSI and Medicaid.
• Simplify rules and application for work incentives for those receiving SSI and/or SSDI.
• Promote awareness of SSI rules among parents when their child(ren) become employed.
Poverty and Financial Empowerment^a^
• Implement wrap-around service models, such as the Integrated Resource Team approach, to address resource needs for people with disabilities from marginalized groups who are experiencing poverty, including issues related to housing/homelessness.
• Advocate for universal healthcare, including access to preventive care and medications.
• Provide training for VR counselors, workforce development professionals, and legislators on the impacts of poverty, including scarcity and trauma-informed approaches.
Discrimination and Stigma
• Implement and enforce policies that prohibit the segregation of people with disabilities, ensuring equal access and opportunities; eliminate allowances and incentives for segregated employment settings and sub-minimum wages; provide grant opportunities to transition away from segregated employment.
• Enforce existing anti-discrimination laws and policies.
• Enhance recognition for employers who develop innovative employment solutions and opportunities for people with disabilities from marginalized groups.
• Showcase successful people with disabilities from marginalized groups in the workplace to combat stigma and discrimination.
Telework
• Foster greater partnerships between VR and Workforce Centers collaborating to offer free, accessible computer skills training and support.
• Provide low-cost internet service for marginalized communities, including rural areas, as well as affordable equipment purchases (e.g., computers).
• Develop policies that focus on expanding access to affordable technology and reliable internet connection in rural and marginalized communities to enable people with disabilities to access online resources, telehealth services and remote work opportunities.
• Develop policies that provide more opportunities for telework, particularly for people with disabilities, addressing telework accessibility and inclusivity.
Vocational Rehabilitation (VR) Eligibility Requirements^a^
• Simplify the VR eligibility determination that requires documentation of disability or diagnosis and the individual's employment history.
• Conduct new VR intakes focused on strengths, interests and preferences as opposed to barrier or deficits.
• Create separate eligibility determination units where VR counselors determine eligibility and provide basic information.
Transportation
• Increase funding for non-profit organizations to maintain transportation services and provide a salary for drivers.
• Increase access to affordable public transportation and rideshare options, potentially by adding more public transportation, larger vehicles, and additional routes to under-served areas.
• Advocate for better funding and equitable transportation options in rural and marginalized communities.
• Develop cross-agency policies to coordinate transportation efforts and establish regional or local councils, involving Vocational Rehabilitation (VR), to explore and develop transportation options in rural areas.
Employer Incentives to Hire People with Disabilities
• Examine the distribution of marginalized populations served by VR agencies to ensure that paid work experience opportunities align with their needs and goals.
• Modify incentive programs to reward forward-thinking hiring practices and sustained employment of people with disabilities.
• Develop incentive programs that are inclusive of various industries and business sizes, ensuring that all sectors have the opportunity to benefit from hiring people with disabilities.
Changes in the Employment Landscape Due to COVID-19
• Expand digital literacy training and increase funding for access to computers, broadband, assistive technology, and accommodations for remote communications and allocate funding to purchase equipment, materials, or supplies needed for individuals to telework in the event of a national or worldwide crisis.
• Explore the development of Career and Technical Education (CTE) programs that cater to the needs and preferences of people with disabilities from marginalized groups in the context of remote work.
• Establish policies to require accommodations for employees with disabilities even during a pandemic or public health emergency.
• Provide education and training regarding accommodations, emphasizing the need for ongoing adjustments that may need to be made to accommodate individuals effectively.
• Create policies to provide accessible communication platforms, aids, devices, and software to employees who require them; provide Wi-Fi hotspots for those without internet access to apply for positions.

^a^ Factor added by Delphi participant (others provided by researchers).

### Emergency preparedness training (median importance score: 4.118)

This participant-added factor was determined to be the most important by all participants. The recommendations emphasized the need for emergency evacuation plans and training that consider the needs of people with disabilities and underscored the importance of safety and preparedness in employment settings. As one participant stated:

“*Not having proper emergency evacuation plans and protocols that consider individuals with disabilities can put their safety at risk during emergencies. During emergencies such as fires, earthquakes, or other incidents requiring evacuation, individuals with disabilities may face challenges due to inaccessible evacuation routes, stairs-only exits, or lack of provisions for those with mobility impairments. Without proper planning, they could be stranded in inaccessible areas, compromising their safety*.”

### Education and training (median importance score: 4.029)

Participants stressed the importance of non-traditional, skills-oriented employment education in schools, with an increased emphasis on apprenticeships, non-college skills, creating portfolios, etc. One participant also indicated the need for:

“*More training in basic skills for life. Teach young people how to use technologies in a redeemable way, instead of giving people phones to dive into TikTok, teach them how to browse and search [for jobs] in Indeed*.”

The recruitment of HR professionals knowledgeable about disability issues emerged as a top recommendation:

“*I would like to see more HRs who are disability community-friendly and can look into other ways to measure qualifications, such as portfolios. It would be great to also change the language of qualifications to where the person can have the opportunity to bring lived experience, learning and experience from other places, volunteer experience, and things outside of degrees to the table*.”

Further, the importance of having programs with expert staff and lived experiences as a person from a multiply marginalized group was emphasized:

“*Require that programs providing education and [employment] training have mentors from and trained to support individuals from marginalized groups*.”

### Assistive technology (AT; median importance score: 4.000)

There was a consensus on allocating more funding to AT programs and integrating AT into educational settings and job training programs early and often. AT emerging as the third most important factor illustrates participants’ recognition of AT's significance in supporting workplace participation for people with disabilities. As one participant's quote illustrates:

“*Invest in assistive technology at all levels to expose youth and adults with disabilities and professionals at all levels.*”

### Accommodations (median importance score: 4.000)

Providing reasonable accommodations in the workplace whenever and wherever they are needed allows people with disabilities equity in the employment landscape and is required by law. As one participant stated:

“*Accommodations can be dismissed as ’special needs’ or ’special privileges,’ though accommodations are necessities to succeed in the workplace..and prejudice against disabled people of color because they are expected to ‘work past their disability’ rather than get accommodations is a big [problem]*.”

Participants also reported that it is essential for VR counselors to be fully aware of accommodations across various sectors. They should discuss reasonable accommodations with the individuals they serve and with employers, who sometimes still do not understand these accommodations. As one participant mentioned:

“*Policy-wise, I think that VR should be educated on accommodations [current, updated tech(nology), etc.] so they know what is available to help their clients and employers*.”

### Complex rules surrounding disability-related benefits (median importance score: 4.000)

Policy changes to simplify rules and procedures related to disability benefits were identified as a key factor added by study participants. Suggested changes included:

“*Simplified rules regarding work incentives for those in receipt of SSI and SSDI; Elimination of waiting periods for cash and medical benefits; Raise income and asset caps to more than 250% of the FPL for SSI and Medicaid; and Allowances for VR to provide support to appeal overpayments*.”

Making disability benefit processes and applications more accessible and understandable was recommended by many participants.

### Poverty, scarcity, and financial empowerment (median importance score: 4.000)

Recommendations for this participant-added factor emphasized the need for wrap-around service models to address the multifaceted needs of people with disabilities experiencing poverty, highlighting a holistic approach to financial empowerment and stability. As one participant noted:

“*Poverty, like disability, is a complex multidimensional phenomenon that cannot be defined by any single factor but instead is an interconnected set of elements (i.e., geographic area, SES, race, gender identity, education level, dis/ability, etc.) and the relationship between disability, poverty, and health is interconnected..[the need for] universal healthcare, access to preventive services and medications.. training for VR counselors and other workforce development professionals on the impacts of poverty, including scarcity and strategies to support individuals with disabilities from multiply marginalized population*.”

### Discrimination and stigma (median importance score: 3.941)

Recommendations related to implementation and enforcement of policies to prohibit disability segregation were stated by several participants, such as:

“*End all allowances and incentives for segregated employment settings and end sub-minimum wages and provide grant opportunities to organizations to end their segregated employment services by a set date*” and “*Anti-discrimination policies already exist. Maybe organizations could expand their policies or diversity trainings on how to identify and address multiple forms of discrimination based on the intersectionality of disability and marginalized groups*.”

Overall, participants indicated fostering an inclusive work environment and combating discrimination by going beyond traditional DEI hiring or initiatives and employers providing education and training led by people with disabilities from marginalized groups and by highlighting success stories.

### Telework (median importance score: 3.941)

The potential of telework was highlighted with recommendations to develop policies to enhance telework opportunities for people with disabilities and funding such initiatives more thoroughly. As one participant suggested:

“*Increase funding for access to computers, broadband, assistive technology and accommodations.. for telework for people with disabilities*.”

### Vocational rehabilitation (VR) eligibility requirements (median importance score: 3.941)

Participants added this factor emphasizing the need to simplify the VR eligibility determination process to be more accessible and user-friendly. Another important detail shared regarding VR eligibility was a recommendation to move away from a deficit-based determination:

“*Eligibility for the public vocational rehabilitation program is predicated on an individual*’*s disability and whether it presents as an impediment to employment. This translates to an eligibility process that determines eligibility by looking at all of the things an individual with a disability cannot do, meaning the process takes a deficit-based approach with the focus on barriers*.”

### Transportation (median importance score: 3.906)

Enhanced funding for non-profit organizations to maintain and expand transportation services was strongly advocated. Additionally, the need for equitable transportation options in rural and marginalized communities highlighted transportation's critical role in successful employment outcomes. Specifically, one participant shared:

“*The Navajo Nation is over 27,000 square miles and the lack of transportation for people with disabilities is a major issue. There is public transportation, but the buses don't have wheelchair lifts or accommodations. It would take over two hours or more to travel to one destination and back. Public transportation doesn't offer a lot of places to stop at and only goes to limited locations. About 40,000 to 50,000 tribal members are impacted with an estimated 30% of adults and 70% of elders have some kind of disability*.”

### Employer incentives to hire people with disabilities (median importance score: 3.824)

In general, we found that even some of the experts on the Delphi panel were not aware of existing incentives available to employers, which may reflect the relatively low importance placed on this factor. They suggested a need for incentives while seemingly unaware that certain tax incentives at the state or local level exist. Among those who were aware of existing incentives, there was a focus on modifying programs to encourage hiring practices that support sustained employment for people with disabilities. As one participant said, “*I'm not sure how well incentives work.*”

The need to provide training about incentives was emphasized. Recommendations for who should conduct such trainings included VR and small business development partnerships that exist in communities across the country. As one participant suggested:

“*Continue to provide pressure on our elected officials and businesses to educate them on the positives on hiring people with disabilities*.”

### Changes in the employment landscape due to COVID-19 (median importance score: 3.558)

Participants highlighted the need for expanded digital literacy training and increased funding for remote work resources. One participant shared:

“*[There is] limited access to technology, high speed internet [and] lack of digital skills for remote work. Many worked in jobs where they could not work from home and had higher risk of exposure*.”

Establishing clear policies and guidance were also recommended moving forward:

“*There may need to be more specifics in place via ADA employment case law regarding what*’*s equitable when in a work-from-home situation versus an office setting. [For example], someone with CP [was] denied telework once COVID was over, even though still able to perform all functions of the job from home*.”

### Further comments

Finally, in all rounds of the Delphi study participants were given space to provide further comments if they thought something of importance was missing or needed further explanation. While only a few participants had additional comments, the following comments provide valuable viewpoints and context:

“*[VR counselors] often end up acting as gatekeepers. And we already know that the majority of special educators and VR counselors are white, often do not have a disability themselves, or do not disclose having one, nor are a member of another marginalized community, my concern is further marginalization of participants with intersectional identities*.”

“*Coming from a sovereign nation we rely on our Tribal Council to make decisions (pass legislations) on behalf of our people. Our leaders aren't very receptive to the needs of people with disabilities when it comes to programs and services to assist with employment. Our tribal government has not adopted ADA rules and regulations and in a perfect world, our tribal leaders would make issues around education and employment a priority. Sovereignty for tribes includes the right to establish their own form of government, determine membership requirements, enact legislation, and establish law enforcement and court systems*.”

## Discussion

The findings from this Delphi study provide critical insights into the multifaceted employment challenges faced by multiply marginalized people with disabilities. These individuals encounter a complex interplay of barriers, influenced by their intersecting identities, that significantly impede their access to and retention of employment. The study's outcomes underscore the necessity for a holistic approach to policymaking and practice that acknowledges and addresses these intersecting barriers.

Interestingly, across most of the various factors, much of the emphasis from participants was on increasing knowledge and providing education—not just for job seekers, but for employers, other employees, and a variety of professionals—on a wide range of issues including emergency preparedness, assistive technology, accommodations, poverty issues, and telework. While some of these factors and recommendations may have been prompted by changes in the workplace due to the pandemic, they also demonstrate an underlying need to use information and training to address long-standing inequities. These findings thus align with the broader literature, which suggests that education and awareness are pivotal in dismantling stereotypes and misconceptions about people with disabilities and supporting improved outcomes (9, 11, 18). Disability policymakers should consider developing new training programs, service initiatives, and research priorities or strategies that enhance the capacity of job seekers, employers, and employment support service professionals to address emergency preparedness, assistive technology, accommodations, economic challenges, and telework needs.

Another broad theme emerging from the study is that many factors external to employment itself play a strong role in whether employment efforts are successful. For example, participants noted the need for wraparound service models that address not only job-readiness, but also issues such as housing, transportation, access to health care, and availability of technology and internet services. Too often, job services and supports focus only on job skills and not the larger context and circumstances of the jobseeker. Again, previous studies have noted the importance of addressing these ostensibly external factors in improving employment for people with disabilities (19–21). In light of this key finding, public employment and vocational rehabilitation service provider systems might consider new initiatives that promote culturally appropriate wraparound services that meet the holistic employment needs of multiply marginalized people with disabilities. Social determinants of health that include housing, transportation, access to health care, and assistive technology have also been found as important for supporting employment aspirations for members of these multiply marginalized disability populations (5, 22).

What sets this study apart is the provision of a list of clear, targeted strategies that offer an actionable path forward for policymakers and practitioners. Unlike previous research that often provides general recommendations, this study identifies specific, actionable strategies as outlined in Table 3. These recommendations are designed to be immediately implementable, offering practical steps that can be taken to address the unique challenges faced by multiple marginalized people with disabilities.

The strategic insights and recommendations gathered from this Delphi study call for a multi-dimensional approach to enhancing employment for multiply marginalized people with disabilities. This approach must be adaptable, responsive to the evolving employment landscape, and grounded in an understanding of the specific barriers these individuals face. However, implementing these strategies involves navigating several potential challenges, such as achieving engagement from vested parties, addressing funding limitations, overcoming policy barriers, and shifting cultural attitudes. Practical guidance for vested parties includes fostering collaboration and networking, identifying and leveraging funding opportunities, advocating for supportive policy changes, providing ongoing education and training, creating inclusive environments, ensuring accessibility, and addressing cultural and systemic barriers to foster equitable employment opportunities.

The consensus reached by experts in this study not only provides specific recommendations for future policy and practice, but also underscores the importance of continued dialogue and collaboration among vested parties to create an inclusive and equitable employment environment for all. Many of the recommendations provided have been posited and discussed for years, yet they persist, and, in some instances, can be viewed as worse since the COVID-19 pandemic affected employment for everyone.

### Limitations

While our Delphi study yields understanding into the employment landscape for multiply marginalized persons with disabilities, it also presents limitations to consider. One limitation is the study's reliance on the knowledge and opinions of experts which, despite being informed and valuable, may not capture the full spectrum of experiences and perspectives of multiply marginalized people with disabilities. In addition, the qualitative nature of the Delphi process, while instrumental in generating rich, detailed insights, also means that the findings may not be generalizable to all settings or populations. Although we engaged a diverse group of experts and vested parties from various cultural, socioeconomic, and geographical backgrounds, future research should consider replicating this study in different contexts and populations to enhance generalizability. This replication could involve adapting the Delphi process to include ever broader representation and testing the recommendations in various settings to assess their applicability and refine strategies to better suit local conditions. Additionally, incorporating longitudinal studies to track the implementation and outcomes of these recommendations across different populations could provide deeper insights into their effectiveness and adaptability.

Another significant limitation is participant attrition over the course of the Delphi process. Starting with 20 participants in Round 1, there were two individuals who were unable to complete Rounds 2 and 3, and an additional participant who did not complete Round 3, leaving a final sample size of 17. This attrition could potentially impact the breadth of perspectives and the robustness of the consensus achieved. The loss of participants might skew the findings, as the remaining panel may not fully represent the diversity of views initially intended. However, retention rates of 85% are considered robust and are unlikely to skew results (23).

The evolving nature of employment, particularly in the post-public health emergency landscape, means that the challenges and opportunities identified may shift over time, requiring ongoing research and adaptation of strategies. Continuous adaptation and testing of the study's insights in response to the changing economic, technical, and social conditions are essential. For instance, exploring the impact of emerging remote work trends and digital divides in different regions can provide a more comprehensive understanding of the employment barriers faced by multiply marginalized people with disabilities.

While the study aims to address the intersecting barriers faced by multiply marginalized people with disabilities, the complexity of these intersections means that not all nuances may be fully explored or understood within the scope of this research. Future Delphi studies could specifically explore the employment challenges of people with disabilities from specific marginalized groups (e.g., Black, LGBTQ+, rural), rather than focusing on multiply marginalized people with disabilities as a whole. Lastly, while the study offers strategic recommendations for improving employment outcomes for people with disabilities from multiply marginalized groups, the implementation of these strategies involves complex systems, which may pose challenges in translating findings into actionable change.

## Conclusion

This Delphi study provides insights into the employment challenges experienced by multiply marginalized people with disabilities and offers strategic policy recommendations for change. This research underscores the necessity for vested parties to adopt and implement its recommendations proactively, emphasizing that improving employment outcomes for people with disabilities not only benefits individuals but augments the entire workforce and economy as a whole. Unlike previous research that often provides general recommendations, this study identifies specific, actionable strategies that offer a clear path forward for policymakers and practitioners. The current findings are important for catalyzing change that has not yet fully materialized despite extensive work and funding over past decades, thereby creating a significantly improved employment outlook for members of these underserved disability populations.

## Data Availability

The raw data supporting the conclusions of this article will be made available by the authors, without undue reservation.
